# Cynanche Trachealis

**Published:** 1829

**Authors:** 


28. *Cynanche Trachealis.*—Assistant professor Jackson of the University of Pennsylvania, in the Am. Jour. for Aug. 1829, has published the histories of three cases of croup, two of which proved fatal, and gave rise to autopsic examinations. The ingenious and indefatigable author concludes his paper in the subsequent language:

The preceding cases justify the following conclusions:—

1st. That inflammation of the tonsils is a frequent precursor to croup in children; and that it ought consequently always, in them, be attentively watched, and should be treated very actively by the means best adapted to reduce imflammation.

2d. That the membranous exudation may commence in the lower part of the trachea and the bronchia, extending upwards, and does not invariably arise in the fauces or larynx, and proceed downwards.

3d. That the most prompt and decisive remedy for sanguine inflammation—that is, sanguineous depletion—is the only certain remedy, and should be resorted to in the first periods of the disease, so as to precede the membranous exudation.

4th. That the membranous exudation having been once produced to any extent, little expectation of a recovery is to be entertained. When this result does occur, it is to be regarded as fortuitous, depending on some extraordinary circumstance, and which cannot be calculated as a probable event.

5th. That when the membranous exudation has been thrown out in the larynx, trachea, and bronchia, the application of muriatic acid to the fauces, so highly extolled by Bretonneau, and of nitrate of silver, recommended by Dr. Mackenzie, from the local and limited impression they must necessarily make, can promise no beneficial operation—their influence cannot be extended to the surfaces from which the exudation takes place. This practice can be of utility only in rare cases, where the difficulty arises from an obstruction limited to the glottis and fauces.

6th. That laryngotomy, or bronchotomy, is a useless operation, when the membranous exudation has actually occurred, and extends as it mostly does, to the trachea and bronchia, or when the inflammatory irritation of the respiratory mucous membrane is not subdued, and a free secretion from it is produced. The impossibility of coughing after the operation, prevents expectoration, the fluids accumulate in the trachea, bronchia, and bronchial ramifications in the lung, causing finally, suffocation. The only case in which the operation promises success, is an obstruction confined entirely to the glottis, without disease prevailing to any extent in the respiratory mucous membrane.—369.

We take this opportunity to remark, that our experienced and judicious friend professor Richardson, of the obstetrical chair in Transylvania University, has long taught in his lectures, that in severe cases of croup, the inflammation extends throughout the ramifications of the bronchia, and is by no means confined to the larynx.

